# Acupuncture for the Treatment of Diarrhea-Predominant Irritable Bowel Syndrome: A Pilot Randomized Clinical Trial

**DOI:** 10.1001/jamanetworkopen.2022.48817

**Published:** 2022-12-29

**Authors:** Ling-Yu Qi, Jing-Wen Yang, Shi-Yan Yan, Jian-Feng Tu, Yan-Fen She, Ying Li, Li-Li Chi, Bang-Qi Wu, Cun-Zhi Liu

**Affiliations:** 1 International Acupuncture and Moxibustion Innovation Institute, School of Acupuncture-Moxibustion and Tuina, Beijing University of Chinese Medicine, Beijing, China; 2 School of Acupuncture-Moxibustion and Tuina, Hebei University of Traditional Chinese Medicine, Shijiazhuang, China; 3 School of Graduate, Chengdu University of Traditional Chinese Medicine, Chengdu, China; 4 Department of Spleen and Stomach, the Affiliated Hospital of Shandong University of Traditional Chinese Medicine, Jinan, China; 5 National Acupuncture and Moxibustion Clinical Medical Research Center, the First Teaching Hospital of Tianjin University of Traditional Chinese Medicine, Tianjin, China

## Abstract

**Importance:**

Acupuncture is a promising therapy for irritable bowel syndrome (IBS), but the use of subjective scales as an assessment is accompanied by high placebo response rates.

**Objectives:**

To preliminarily test the feasibility of using US Food and Drug Administration (FDA)–recommended end points to evaluate the efficacy of acupuncture in the treatment of IBS.

**Design, Setting, and Participants:**

This pilot, multicenter randomized clinical trial was conducted in 4 tertiary hospitals in China from July 1, 2020, to March 31, 2021, and 14-week data collection was completed in March 2021. Individuals with a diagnosis of IBS with diarrhea (IBS-D) were randomized to 1 of 3 groups, including 2 acupuncture groups (specific acupoints [SA] and nonspecific acupoints [NSA]) and a sham acupuncture group (non-acupoints [NA]) with a 1:1:1 ratio.

**Interventions:**

Patients in all groups received twelve 30-minute sessions over 4 consecutive weeks at 3 sessions per week (ideally every other day).

**Main Outcomes and Measures:**

The primary outcome was the response rate at week 4, which was defined as the proportion of patients whose worst abdominal pain score (score range, 0-10, with 0 indicating no pain and 10 indicating unbearable severe pain) decreased by at least 30% and the number of type 6 or 7 stool days decreased by 50% or greater.

**Results:**

Ninety patients (54 male [60.0%]; mean [SD] age, 34.5 [11.3] years) were enrolled, with 30 patients in each group. There were substantial improvements in the primary outcomes for all groups (composite response rates of 46.7% [95% CI, 28.8%-65.4%] in the SA group, 46.7% [95% CI, 28.8%-65.4%] in the NSA group, and 26.7% [95% CI, 13.0%-46.2%] in the NA group), although the difference between them was not statistically significant (*P* = .18). The response rates of adequate relief at week 4 were 64.3% (95% CI, 44.1%-80.7%) in the SA group, 62.1% (95% CI, 42.4%-78.7%) in the NSA group, and 55.2% (95% CI, 36.0%-73.0%) in the NA group (*P* = .76). Adverse events were reported in 2 patients (6.7%) in the SA group and 3 patients (10%) in NSA or NA group.

**Conclusions and Relevance:**

In this pilot randomized clinical trial, acupuncture in both the SA and NSA groups showed clinically meaningful improvement in IBS-D symptoms, although there were no significant differences among the 3 groups. These findings suggest that acupuncture is feasible and safe; a larger, sufficiently powered trial is needed to accurately assess efficacy.

**Trial Registration:**

Chinese Clinical Trial Registry: ChiCTR2000030670

## Key Points

**Question:**

Is the US Food and Drug Administration (FDA)–recommended end point feasible in clinical trials of acupuncture for irritable bowel syndrome (IBS)?

**Findings:**

In this pilot randomized clinical trial of 90 patients, the use of an FDA-recommended end point as the primary outcome was feasible for clinical trials of acupuncture for IBS. No significant between-groups differences in IBS symptom improvement were found.

**Meaning:**

The findings of this pilot trial may provide a more accurate basis for assessing the efficacy of acupuncture for IBS in subsequent clinical trials.

## Teaser

This pilot randomized clinical trial of patients with irritable bowel syndrome with diarrhea examines whether the use of US Food and Drug Administration–recommended end points is feasible for determining the efficacy of acupuncture.

## Introduction

Irritable bowel syndrome (IBS) is a common disorder of gut-brain interaction characterized by abdominal pain associated with defecation or changes in bowel habits.^1^ The condition affects approximately 5% to 10% of individuals in most geographic regions^2^ and is 1 of the top 10 reasons for seeing a primary care physician.^3,4,5,6^ Patients with IBS report worse health-related quality of life than patients with diabetes or end-stage kidney disease^7,8^ and even are willing to accept a 1% median risk of sudden death in exchange for a 99% chance of using hypothetical medications to cure symptoms.^9^ Available treatments target IBS symptoms rather than underlying pathophysiological mechanisms, and additional improvements in the testing methods are still necessary.^10^ All these factors have resulted in an increasing interest in complementary and alternative medicine, such as acupuncture.^11,12,13,14^

A previous study^15^ suggested that acupuncture has promising effects on IBS. The possible biological mechanisms that have been proven to be involved in acupuncture for IBS primarily included reducing visceral hypersensitivity and modulating the gut-brain axis.^16^ However, clinical trials in IBS are associated with high placebo response rates, especially when choosing the subjective scale as the primary end point.^17^ Recent evidence^18^ suggested that future randomized clinical trials should adhere to current US Food and Drug Administration (FDA)–recommended composite end points for IBS, which leads to lower placebo response rates. Therefore, the first aim of the current study was to preliminarily test the feasibility of using FDA-recommended end points to evaluate the efficacy of acupuncture treatment for IBS.

The acupoint is considered one of the most determining factors in the efficacy of acupuncture, and the choice of more appropriate acupoints for stimulation is meaningful for acupuncture clinical application. According to traditional meridian and acupoint theories, acupoints were divided into the specific acupoint (SA) and the nonspecific acupoint (NSA), and the selection of specific points can result in greater efficacy. However, no studies have specifically addressed the efficacy of specific acupoint programs. Therefore, the second aim of this study was to compare the difference in acupuncture efficacy in patients receiving SA treatment, NSA treatment, or nonacupoint (NA) treatment.

## Methods

### Study Design

There is likely considerable uncertainty when preparing a large-scale trial to evaluate an intervention using primary outcomes that are not widely adopted, and therefore feasibility and safety need to be tested and demonstrated before committing considerable human and monetary resources preliminarily.^19^ The current study was a pilot, multicenter randomized clinical trial with 2-week screening, 4-week treatment, and 8-week follow-up for patients with IBS. The protocol and statistical analysis plan have been previously published and are provided in Supplement 1.^20^ We recruited patients from 4 tertiary hospitals in China from July 1, 2020, to December 31, 2020, and 14-week data collection was completed in March 2021. This study was approved by the ethics committees of Beijing University of Chinese Medicine and each study site and followed the Consolidated Standards of Reporting Trials (CONSORT) reporting guideline. Informed consent was provided by all patients before randomization.

### Patients

Chinese patients aged 18 to 75 years who met the Rome IV criteria for diarrhea-predominant IBS (IBS-D) were included in this study of acupuncture treatment of diarrhea-predominant IBS. During the 2-week screening, eligible patients were defined as those whose type 6 or 7 stools of the Bristol Stool Form Scale appeared for at least 4 days and type 1 or 2 stools appeared for less than 4 days, and the mean score of daily abdominal pain was 3 or higher in the last week. The exclusion criteria primarily ruled out patients with organic gastrointestinal disease (eg, inflammatory bowel disease, microscopic colitis, celiac disease, and Crohn disease), and some patients (those ≥50 years of age or who had the following alarm signs: unexplained weight loss [weight loss >10% within 3 months], hematochezia caused by nonhemorrhoids or anal fissure, nocturnal diarrhea, or family history of colorectal cancer) were required to provide normal results of endoscopy within 2 years before study entry.

### Randomization and Masking

Patients were randomized to 1 of the 3 trial arms (SA group, NSA group, and NA group) according to the ratio of 1:1:1. Randomization was stratified by recruitment site, with a fixed block size of 6. An independent statistician who was not involved in the implementation of statistical analysis generated the blocked randomization sequence by using SAS software, version 9.3 (SAS Institute Inc). The randomization sequence was stored by the special randomization sequence manager, and the clinical research coordinators obtained the randomization number through a telephone randomization process. The acupuncturists could not be masked in treatment allocation. Patients, clinical recruiters, outcome assessors, data managers, and statisticians were blinded.

### Interventions

Patients in all groups started treatment on the day of randomization and received twelve 30-minute sessions over 4 consecutive weeks at 3 sessions per week (ideally every other day). The treatments were administered by certified acupuncturists who had 5 years of undergraduate education in acupuncture and at least 3 years of clinical experience. Each acupuncturist received a 2-day training and could perform treatments for all groups, with priority given to the same acupuncturist delivering treatment to a specific patient throughout the trial whenever possible. Single-use sterile needles (length: 25 to 40 mm; diameter: 0.30 mm; Hwato) were used in acupuncture groups. Blunt-tipped placebo needles with similar appearances to conventional needles but no skin penetration were used in the sham acupuncture group. Adhesive pads were placed on acupuncture points in all groups, which is to help maximize the blinding of patients in the NA group and to fix blunt-tipped placebo needles (eFigure 1 in Supplement 2).

Patients in the SA group received acupuncture at 6 acupoints (5 fixed acupoints and 1 of 3 optional acupoints) according to the syndrome diagnosis and the principle of matching specific acupoints. The 6 fixed acupoints of the NSA group were chosen based on the frequency of acupoint use that excluded the acupoints in the SA group. Insertion was followed by stimulation performed by lifting and thrusting the needle combined with twirling and rotating the needle sheath to produce *deqi* (sensation of soreness, numbness, distention, or radiating).^21^ Five nonacupoints away from meridians or conventional acupoints were selected in the NA group without manipulations. The locations of acupoints in each group were described in our study protocol.^20^ Loperamide was used as rescue medication, and the patients were encouraged to refrain from using medications or other therapies for the management of IBS throughout the trial.

### Outcomes

The composite response rate at week 4 of the treatment phase was chosen as the primary outcome. According to FDA recommendations, eligible composite responders responded in both abdominal pain intensity and stool consistency, defined as at least a 30% decrease in the weekly mean of worst abdominal pain in the past 24 hours compared with baseline and a 50% or greater reduction in the number of days per week with at least 1 stool that has a consistency of type 6 or 7 compared with baseline.

Secondary outcomes included a composite response rate of other time points, IBS Symptom Severity Scale (IBS-SSS), IBS–Quality of Life scale (IBS-QOL), Patient Health Questionnaire 9 (PHQ-9) depression scale, IBS Adequate Relief (IBS-AR), and IBS-D individual symptoms (abdominal pain, bloating, loose stool day, and stool frequency recording on defecation diaries [eFigure 2 in Supplement 2]). A 50-point decrease in IBS-SSS score is adequate to reliably indicate clinical improvement, and meaningful clinical response of IBS-QOL is represented by an increase of at least 14 points. For IBS-D individual symptoms, abdominal pain and bloating were assessed by a 0- to 10-point visual analogue scale, with 0 indicating no pain and 10 indicating unbearable severe pain, and the Bristol Stool Score was used to record loose stool days and stool frequency. Patients were asked to complete the credibility and expectancy questionnaire 5 minutes after the first treatment and were asked to guess which treatment they had received to test whether the blinding was successful after the last treatment (see protocol for details and eFigure 3 in Supplement 2).

The researchers in charge of the scale assessment were asked to use the fixed guiding words on the questionnaires to have a conversation with the patient without too much communication. Due to the trial site and population, we used Chinese versions of the assessment scales that were confirmed to have moderate or higher clinical responsiveness and are suitable for clinical efficacy evaluation.^22,23,24^

### Statistical Analysis

According to the method of upper confidence limit, a sample size ranging from 20 to 40 can be the guideline for choosing the size of a pilot sample. Considering the overall resource input issues (eg, funding availability and expected completion time),^25^ the total sample size was fixed at 90 patients, 30 patients per group. An intention-to-treat set was used in all efficacy analyses, and the missing data were imputed using the last observation carried forward. Relevant data were summarized with numbers (percentages) for categorical data and means (SDs) or medians (IQRs) for continuous data.

For the primary outcome, a logistic generalized linear mixed model that included baseline abdominal pain score and loose stool days as covariates were used, with time and group as fixed factors, patient as a random factor, and logit function set as the link function. For sensitivity analysis, a per-protocol analysis was used for the primary outcome, covering patients who complete 10 sessions or more and who had no major protocol violations.

For change scores of PHQ-9 and IBS-D individual symptoms, an analysis of variance was used for comparison among the 3 groups. The response rates at other time points, IBS-SSS, IBS-QOL, IBS-AR, blinding assessment, and adverse event rates were analyzed using the χ^2^ test or Fisher exact test. Analyses were performed with SPSS software, version 22.0 (IBM Inc). All reported *P* values are 2-sided with a significance level of <.05.

## Results

### Patients and Characteristics

A total of 201 patients with IBS-D were screened. Of these, 111 (55.2%) were excluded for various reasons, 90 (44.8%) patients (54 male [60.0%] and 36 female [40%]; mean [SD] age, 34.5 [11.3] years) were enrolled and randomized (Figure), and 11 patients (12.2%) dropped out. There was no difference among the study groups in the number of patients lost to follow-up (eTable 1 in Supplement 2). All clinical and baseline demographic characteristics were balanced in the 3 groups (Table 1).

**Figure.**
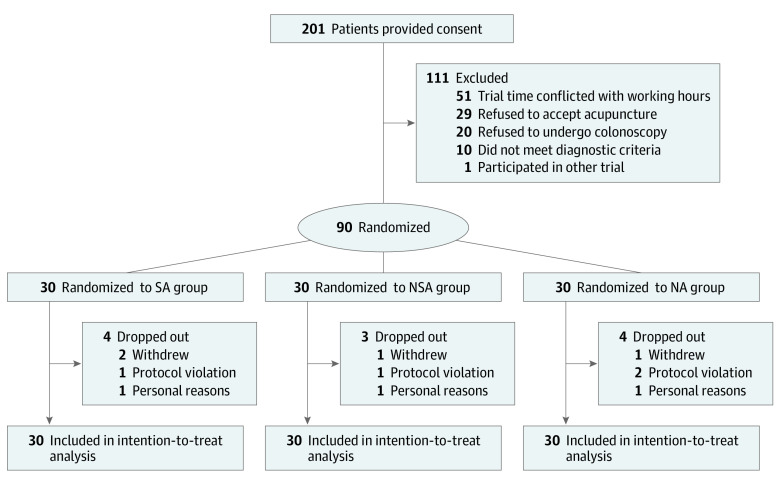
Trial Flow Diagram NA indicates nonacupoint; NSA, nonspecific acupoint; and SA, specific acupoint.

**Table 1.** Baseline Characteristics of the Intention-to-Treat Population^a^

Characteristic	SA (n = 30)	NSA (n = 30)	NA (n = 30)
Age, y	36.7 (12.2)	31.0 (9.9)	35.7 (11.1)
Sex, No. (%)			
Female	11 (36.7)	10 (30.0)	15 (50.0)
Male	19 (63.3)	20 (70.0)	15 (50.0)
BMI	24.0 (4.1)	22.9 (3.7)	22.3 (3.4)
IBS-D course, y	6.5 (2.9)	8.2 (4.1)	7.7 (3.6)
Occupation, No. (%)			
Mental	26 (86.7)	27 (90.0)	27 (90.0)
Manual	4 (13.3)	3 (10.0)	3 (10.0)
IBS-SSS score	271.5 (63.0)	281.0 (77.7)	274.5 (84.1)
IBS-QOL score^b^	74.0 (13.9)	68.5 (18.2)	73.9 (14.3)
PHQ-9 score	8.2 (5.5)	8.0 (4.7)	7.5 (4.6)
Loose stool days	5.3 (1.4)	5.4 (1.7)	5.3 (1.6)
Abdominal pain score	4.6 (1.6)	4.3 (1.3)	4.3 (1.4)
Bloating score	3.3 (2.1)	3.5 (1.9)	3.6 (2.0)
Stool frequency	2.5 (1.2)	2.5 (1.7)	2.6 (1.3)
Credibility^c^	−0.2 (3.3)	0.1 (2.3)	−0.2 (3.3)
Expectancy^c^	−0.7 (3.0)	0.4 (1.9)	−0.1 (3.7)

Abbreviations: BMI, body mass index (calculated as weight in kilograms divided by height in meters squared); IBS, irritable bowel syndrome; IBS-D, IBS with diarrhea; IBS-QOL, IBS–Quality of Life scale; IBS-SSS, IBS Symptom Severity Scale; NA, nonacupoint; NSA, nonspecific acupoint; PHQ-9, Patient Health Questionnaire-9 depression scale; SA, specific acupoint.

^a^ Data are presented as mean (SD) unless otherwise indicated.

^b^ The sum of the IBS-QOL items was transformed into a score, with higher scores indicating better quality of life (100 meaning maximum quality of life).

^c^ Credibility was defined as how believable, convincing, and logical the treatment is, and expectancy refers to improvements that patients believe will be achieved. The scale has a mean (SD) of 0.0 (1.0) because items were converted to *z* scores before averaging. Zero represents the mean level of credibility/expectation around the population. The *z* score is negative when the credibility/expectancy is below the mean and positive when it is above the mean.

### Primary Outcome

The composite response rate at week 4 was 46.7% (95% CI, 28.8%-64.6%) in the SA group, 46.7% (95% CI, 28.8%-64.6%) in the NSA group, and 26.7% (95% CI, 10.9%-42.5%) in the NA group, and no significant difference was found among the 3 groups (Table 2). The results of the per-protocol analysis were similar to the results of the intention-to-treat analysis (53.8%in the SA group, 51.9% in the NSA group, and 30.8% in the NA group). Missing patterns were monotone, and the differences among the study groups were not significant (eTable 2 in Supplement 2).

**Table 2.** Outcomes, Differences Between Groups, and Changes Over Time

Efficacy outcome	SA (n = 30)	NSA (n = 30)	NA (n = 30)	*P* value
**Primary outcome**				
Response rates^a^ at week 4 (95% CI)	46.7 (28.8-65.4)	46.7 (28.8-65.4)	26.7 (13.0-46.2)	.18
**Secondary outcomes**				
Response rates at other time points (95% CI)				
Week 1	26.7 (13.0-46.2)	20.0 (8.4-39.1)	26.7 (13.0-46.2)	.79
Week 2	30.0 (15.4-49.6)	36.7 (20.6-56.1)	30.0 (15.4-49.6)	.82
Week 3	36.7 (20.6-56.1)	46.7 (28.8-65.4)	33.3 (19.9-52.9)	.54
Week 8	53.3 (34.6-71.2)	46.7 (28.8-65.4)	53.3 (34.6-71.2)	.84
Week 12	60.0 (40.8-76.8)	66.7 (47.1-82.1)	50.0 (31.7-68.3)	.42
IBS-SSS response rates (95% CI)				
Week 2	60.0 (40.8-76.8)	63.3 (43.9-79.5)	53.3 (34.6-71.2)	.72
Week 4	70.0 (50.4-84.6)	66.7 (47.1-82.1)	66.7 (47.1-82.1)	.95
Week 8	66.7 (47.1-82.1)	70.0 (50.4-84.6)	63.3 (43.9-79.5)	.86
Week 12	73.3 (53.8-87.0)	76.7 (57.3-89.4)	66.7 (47.1-82.1)	.68
IBS-QOL response rates (95% CI)				
Week 2	16.7 (6.3-35.5)	16.7 (6.3-35.5)	13.3 (4.4-31.6)	.92
Week 4	43.3 (26.0-62.3)	30.0 (15.4-49.6)	26.7 (13.0-46.2)	.35
Week 8	46.7 (28.8-65.4)	33.3 (17.9-52.9)	26.7 (13.0-46.2)	.26
Week 12	43.3 (26.0-62.3)	46.7 (28.8-65.4)	33.3 (17.9-52.9)	.55
Changes in PHQ-9 score, mean (SD)				
Week 2	1.3 (3.9)	1.3 (2.2)	0.4 (2.9)	.45
Week 4	2.7 (4.2)	2.0 (2.8)	1.4 (3.7)	.38
Week 8	3.1 (4.8)	1.9 (3.5)	2.1 (4.0)	.44
Week 12	3.6 (5.4)	2.1 (5.2)	2.5 (3.5)	.42
AR response rates (95% CI)^b^				
Week 1	39.3 (22.1-59.3)	34.5 (18.6-54.3)	55.2 (36.0-73.0)	.25
Week 2	53.6 (34.2-72.0)	51.7 (32.9-70.1)	55.2 (36.0-73.0)	.97
Week 3	53.6 (34.2-72.0)	55.2 (36.0-73.0)	44.8 (27.0-64.0)	.70
Week 4	64.3 (44.1-80.7)	62.1 (42.4-78.7)	55.2 (36.0-73.0)	.76
Week 8	60.7 (40.7-77.9)	41.4 (24.1-60.9)	46.4 (28.0-65.8)	.32
Week 12	60.7 (40.7-77.9)	48.3 (29.9-67.1)	46.4 (28.0-65.8)	.51
Changes in abdominal pain score, mean (SD)				
Week 1	1.2 (2.0)	1.0 (1.1)	1.2 (1.4)	.85
Week 2	1.6 (2.1)	1.3 (1.2)	1.4 (1.3)	.75
Week 3	2.0 (2.1)	1.5 (1.2)	1.7 (1.4)	.44
Week 4	2.2 (2.4)	1.7 (1.2)	1.6 (1.5)	.36
Week 8	2.5 (2.2)	2.1 (1.3)	2.2 (1.3)	.61
Week 12	2.7 (2.2)	2.4 (1.5)	2.5 (1.6)	.82
Changes in loose stool day, mean (SD)				
Week 1	2.2 (2.5)	1.3 (2.1)	1.7 (2.5)	.31
Week 2	2.2 (2.6)	2.0 (2.3)	2.1 (2.4)	.93
Week 3	2.7 (2.4)	2.4 (2.1)	2.7 (2.9)	.83
Week 4	3.1 (2.6)	2.7 (2.6)	2.5 (2.6)	.60
Week 8	3.3 (2.5)	2.6 (2.5)	3.2 (2.9)	.52
Week 12	3.4 (2.6)	3.3 (2.5)	3.2 (2.7)	.96
Changes in bloating score, mean (SD)				
Week 1	0.7 (1.8)	0.9 (1.3)	1.1 (1.5)	.50
Week 2	1.1 (1.8)	1.1 (1.3)	1.5 (1.5)	.50
Week 3	1.3 (1.7)	1.1 (1.3)	1.3 (1.7)	.85
Week 4	1.7 (2.1)	1.4 (1.6)	1.9 (1.5)	.50
Week 8	1.6 (2.1)	1.7 (1.8)	2.1 (1.6)	.62
Week 12	3.3 (2.1)	3.5 (1.9)	3.6 (2.0)	.86
Changes in stool frequency, mean (SD)				
Week 1	0.3 (0.8)	0.2 (0.6)	0.2 (0.5)	.72
Week 2	0.3 (0.7)	0.2 (0.6)	0.1 (0.4)	.42
Week 3	0.4 (0.8)	0.2 (0.5)	0.3 (0.4)	.73
Week 4	0.4 (0.8)	0.3 (0.6)	0.4 (0.5)	.83
Week 8	0.5 (0.8)	0.3 (0.7)	0.4 (0.8)	.56
Week 12	0.5 (1.0)	0.6 (1.0)	0.6 (1.0)	.93

Abbreviations: AR, adequate relief; IBS, irritable bowel syndrome; IBS-QOL, IBS–Quality of Life scale; IBS-SSS, IBS Symptom Severity Scale; NA, nonacupoint; NSA, nonspecific acupoints; PHQ-9, Patient Health Questionnaire 9; SA, specific acupoint.

^a^ Response rate values are the FDA-recommended composite response rates, defined as the proportion of patients whose worst abdominal pain score (range, 0-10) decreased by at least 30% and whose number of type 6 or 7 stool days decreased by at least 50%.

^b^ A total of 4 patients lacked available AR assessment data, so the sample sizes were 28 in the SA group, 29 in the NSA group, and 29 in the NA group.

### Secondary Outcomes

Similar to our primary outcome, the observed levels of improvement across the 3 groups were generally similar whether immediately after the 12 treatments or at follow-up, and the 3 groups did not differ significantly from any of these secondary efficacy outcomes (Table 2; eFigure 4 in Supplement 2). As for the success of blinding, no difference was found among groups in the proportion of patients who guessed that they received acupuncture immediately after the 12th session (eTable 3 in Supplement 2).

### Adverse Events

Two adverse events (6.6%) occurred in the SA group, 4 (13.3%) occurred in the NSA group, and 4 (13.3%) occurred in the NA group. Treatment-related adverse events were mild and transient. No serious adverse events or rescue medication applications were reported (Table 3).^26^

**Table 3.** Adverse Events Related and Unrelated to Treatment

Adverse event^a^	No. (%) of adverse events		
	SA (n = 30)	NSA (n = 30)	NA (n = 30)
Any	2 (6.6)	4 (13.3)	4 (13.3)
Serious	0	0	0
Related to treatment^b^			
Hematoma	0	1 (3.3)	1 (3.3)
Sensation after needle removal	1 (3.3)	2 (6.6)	1 (3.3)
Residual needling	1 (3.3)	0	1 (3.3)
Unrelated to treatment			
Upper respiratory tract infection	0	1 (3.3)	0
Stomach pain	0	0	1 (3.3)

Abbreviations: NA, nonacupoint; NSA, nonspecific acupoint; SA, specific acupoint.

^a^ Adverse events were counted by type rather than the frequency in the same participant. Adverse events of different types occurring in a single participant were defined as independent adverse events. An adverse event with multiple occurrences in a single participant was defined as 1 adverse event.^26^

^b^ A treatment-related adverse event was defined as any adverse event that was related to the trial intervention as determined by acupuncturists and gastroenterologists.

## Discussion

Placebo effects are considered a part of the efficacy of acupuncture,^27^ and clinical trials in IBS are themselves associated with high placebo response rates,^18^ making it difficult to draw appropriate conclusions about the specific efficacy of acupuncture for IBS. A meta-analysis^18^ of 73 IBS randomized clinical trials showed that the magnitude of the pooled placebo response rate in pharmacological trials in IBS was 27.3% for the global improvement responder end point. After the introduction of the composite FDA end point, the pooled placebo response decreased to 17.9%, but the therapeutic gain remained unaltered. The present pilot trial was, to our knowledge, the first parallel, 3-group, multicenter, randomized clinical trial using a prespecified composite response rate recommended by the FDA as the primary outcome in trials on acupuncture treatment of IBS. The enrollment rate of 55.2% and the dropout rate of 12.2% are approximate to previous studies,^28^ and an even higher completion rate (91.1%) of treatment suggests that it is feasible to use the Rome IV diagnostic criteria and FDA-recommended end points in studies of acupuncture for IBS-D.^29^

Several noteworthy findings support the use of SAs. First, a 10% to 15% improvement of the global outcome measure over a placebo could be considered a clinically significant therapeutic gain.^30^ The 20% difference between acupuncture and sham acupuncture suggests that it may have constituted a meaningful outcome in the actual clinical treatment.^31^ Second, IBS-AR correlates with improvement in individual IBS symptoms. After 12 sessions of treatment, the week 4 response rate of IBS-AR in the SA group reached more than 60.0% and was maintained during follow-up, which was similar to a previous study^32^ that concluded intervention medications can effectively improve the symptoms of IBS. However, this response rate gradually decreased to approximately 40% to 50% in the NSA and NA groups. Third, the IBS-QOL response rates at the end of treatment were 10% to 20% higher in the SA group than in the NA group. Last, loose stool days in the SA group decreased more than 3 days at weeks 4, 8, and 12 compared with baseline, which means that patients may no longer fall into the Rome IV definition of the IBS-D disease population due to the improvement in bowel status.

We have considered the reasons for the negative results of our study. First, the small sample size resulted in insufficient power and underestimated the true efficacy. Second, considering the time-economic cost and patient adherence, we have provided only 12 sessions in this pilot trial. Therefore, adding the acupuncture dose by increasing the treatment duration (eg, 16-18 sessions for 5-6 weeks) may be an effective way to optimize the treatment program. Third, the state of *deqi* is a key factor affecting the efficacy of acupuncture. To better fit the actual clinical treatment situation, the frequency of *deqi* operation will be increased; that is, the operation of *deqi* will be performed every 10 minutes during the needle retention time.

In the current study, patients in all 3 groups showed further improvement in the follow-up phase after the end of treatment. We speculate that this finding may be related to the delayed effects of the acupuncture treatment in the acupuncture group and the self-healing process of the disease in the sham acupuncture group. The finding of a nonrepeated, event-related functional magnetic resonance imaging–designed trial showed that the delayed effects may be related to a more significant characteristic of the effect of acupuncture treatment.^33^ Moreover, the placebo treatment has been documented to produce significant improvement in IBS symptoms through mind-body self-healing processes.^34^ In the IBS trials of the TARGET (Rifaximin 3 Times/Day for Non-Constipation Irritable Bowel Syndrome) Study Group,^35^ the response rates of adequate relief in the placebo group exceeded 30%. In addition, in the current study, the blunt-tipped placebo needles used in the NA group cannot pierce the skin but still cause irritation to the skin, which may increase the placebo effect in patients in the NA group. Therefore, a 26.7% response rate in the NA group might be acceptable.

### Strengths and Limitations

This study had several strengths. First, the 2-week screening phase is a critical step to improving the quality of this trial. The inaccuracy of the study population is an important confounding factor affecting the results of the trial. The 2-week screening phase can better distinguish patients with IBS-D via the overall evaluation of the defecation diaries, and approximately 9.0% of screened patients were excluded without passing the 2-week screening phase (Figure).^18^ Second, the use of blunt-tipped placebo needles ensured the implementation of blinding, which can make the patients have the feeling of acupuncture under the premise that the needle tip does not penetrate the skin.^36^ Third, the current study may provide a more accurate basis for assessing the sample size and selection of acupuncture acupoints for the large-scale trial to be conducted.

There are also several limitations to the current study. First, the use of a minimal sample size may increase the risk that a significant treatment benefit will not be shown (a type 2 error), even if such an effect exists.^37^ Second, although we used the composite outcome approved by the FDA for this trial, defecation diaries as the data sources are subjective and susceptible to interference from potential biases of self-reporting. To avoid it, researchers maintained continuous contact with patients and supervised the correct filling of defecation diaries, which aimed to ensure that the defecation diaries of patients are filled out every day as much as possible. Third, because of the low willingness of patients, we did not collect blood, stool, or other specimens in this study, and the mechanism of acupuncture for IBS remains to be further explored. Fourth, acupuncturists could not be blinded, which may affect the effect of interventions between groups.

## Conclusions

The findings of this pilot randomized clinical trial suggest that acupuncture is feasible and safe for the treatment of IBS-D. To accurately assess the efficacy of acupuncture for IBS-D, a larger, sufficiently powered trial with the FDA-recommended composite response rate as the primary outcome is needed.

## Notes

Supplement 1.
Trial Protocol and Statistical Analysis Plan

Supplement 2. **eFigure 1.** The Schematic Diagram of Sham-Acupuncture **eFigure 2.** Defecation Diary Card **eFigure 3.** The Procedure Diagram **eFigure 4.** Schematic Diagram of Changes in Each Outcome During Treatment and Follow-up **eTable 1.** Information on Patients Lost to Follow-up **eTable 2.** Distribution of the Completeness of the Composite Response Rate Data During Weeks 1-4 **eTable 3.** Blinding Assessment

Supplement 3.
Data Sharing Statement
