# Supplementary material for: Acupuncture for the Treatment of Diarrhea-Predominant Irritable Bowel Syndrome: A Pilot Randomized Clinical Trial
Source: JAMA Netw Open. 2022 Dec 29;5(12):e2248817. doi: 10.1001/jamanetworkopen.2022.48817 (PMC9856830; doi:10.1001/jamanetworkopen.2022.48817)
Supplement: Supplement 1. — Trial Protocol and Statistical Analysis Plan [file jamanetwopen-e2248817-s001.pdf]

Acupuncture for the treatment of Diarrhea-predOminant irritable Bowel syndromE (ADOBE): A randomized controlled pilot trial

Study Protocol
Final Version

Clinical site:

Beijing Hospital of Traditional Chinese Medicine, Capital Medical University, Dongcheng District, Beijing, China

Data management and statistical site:

International Acupuncture and Moxibustion Innovation Institute, School of Acupuncture-Moxibustion and Tuina, Beijing University of Chinese Medicine, Chaoyang District, Beijing, China

Data:

Original protocol date: March 9 2020
Amendment date: February 6, 2021

Contents

1 Committee composition 3
1.1 Protocol committee 3
1.2 Steering committee 3
1.3 Data coordination committee 3
1.4 Publication committee 3
2 Study contacts and organizations 3
2.1 Study contacts 3
2.2 Recruiting site 4
3 Background 4
4 Study hypotheses 5
5 Methodology 5
5.1 Study design 5
5.2 Randomization and blinding 5
5.3 Sample size 6
5.4 Participant recruitment, screening and group assignment 6
5.5 Inclusion criteria 6
5.6 Exclusion criteria 7
6 Outcome measurement 7
6.1 Primary outcome 7
6.2 Secondary outcomes 7
7 Interventions 8
7.1 Specific acupoints group 8
7.2 Non-specific acupoints group 9
7.3 Non-acupoints group 10
8 Data management and quality control 10
9 Statistical analysis 11
10 Ethics and dissemination 11
References 12

1 Committee composition

1.1 Protocol committee

Table 1 Protocol committee

Name	Affiliation	Email address
Cun-Zhi Liu	Beijing University of Chinese Medicine	lcz623780@126.com
Ling-Yu Qi	Beijing University of Chinese Medicine	qly66love@163.com
Jing-Wen Yang	Beijing University of Chinese Medicine	yangjw0626@126.com
Shi-Yan Yan	Beijing University of Chinese Medicine	yanshiyan0927@sina.com
Jian-Feng Tu	Beijing University of Chinese Medicine	tujianfeng1@126.com

1.2 Steering committee

Table 2 Steering committee

Name	Affiliation	Email address
Cun-Zhi Liu	Beijing University of Chinese Medicine	lcz623780@126.com
Yan-Fen She	Hebei University of Chinese Medicine	sheyanfen@163.com
Ying Li	Chengdu University of Chinese Medicine	liying@cdutcm.edu.cn
Li-Li Chi	the Affiliated Hospital of Shandong University of Traditional Chinese Medicine	chililiyl@163.com
Bang-Qi Qu	the First Teaching Hospital of Tianjin University of Traditional Chinese Medicine	wbqwbq1980@outlook.com

1.3 Data coordination committee

Table 3 Data coordination committee

Name	Affiliation	Email address
Jing-Wen Yang	Beijing University of Chinese Medicine	yangjw0626@126.com
Shi-Yan Yan	Beijing University of Chinese Medicine	yanshiyan0927@sina.com
Jian-Feng Tu	Beijing University of Chinese Medicine	tujianfeng1@126.com

1.4 Publication committee

Members of the publication committee include Cun-Zhi Liu, Ling-Yu Qi.

2 Study contacts and organizations

2.1 Study contacts

Principal investigator for grant

Cun-Zhi Liu, MD PhD
International Acupuncture and Moxibustion Innovation Institute, School of Acupuncture-Moxibustion and Tuina, Beijing University of Chinese Medicine; No. 11, Bei San Huan Dong Lu, Chaoyang District, Beijing 100029, China
Phone: +86 1053912201
Email: lcz623780@126.com

Principal investigator for trial

Cun-Zhi Liu, MD PhD
International Acupuncture and Moxibustion Innovation Institute, School of Acupuncture-Moxibustion and

Tuina, Beijing University of Chinese Medicine; No. 11, Bei San Huan Dong Lu, Chaoyang District, Beijing 100029, China
Phone: +86 1053912201
Email: lcz623780@126.com

2.2 Recruiting site

The First Affiliated Hospital of Hebei University of Chinese Medicine
Yan-Fen She
School of Acupuncture-Moxibustion and Tuina, Hebei University of Chinese Medicine, Shijiazhuang 050299, China;
E-mail: sheyanfen@163.com.
The Affiliated Hospital of Chengdu University of Traditional Chinese Medicine
Ying Li
School of Graduate, Chengdu University of Traditional Chinese Medicine, Chengdu 610075, China;
E-mail: liying@cdutcm.edu.cn.
The Affiliated Hospital of Shandong University of Traditional Chinese Medicine
Li-Li Chi
Department of Spleen and Stomach, the Affiliated Hospital of Shandong University of Traditional Chinese Medicine, Jinan 250011, China;
E-mail: chililiyl@163.com.
The First Teaching Hospital of Tianjin University of Traditional Chinese Medicine
Bang-Qi Wu
National Acupuncture and moxibustion Clinical Medical Research Center, the First Teaching Hospital of Tianjin University of Traditional Chinese Medicine, Tianjin 300193, China;
E-mail: wbqwbq1980@outlook.com.

3 Background

Irritable bowel syndrome (IBS) is a functional gastrointestinal disease,[1] with abdominal pain, abdominal distension, changes in defecation habits and stool irregularities as the main clinical manifestations[2]. The global prevalence of IBS is approximately 11%,[3] while the prevalence in different regions of Asia ranges from 5.0% to 9.9%.[4] IBS, one of the most common reasons for the abnormal state of life and work,[5] increased the economic burden and impaired the life quality of patients[6,7]. According to the Rome IV criteria,[1] the diarrheal-predominant IBS (IBS-D) is the most frequently occurring subtype accounting for 40%.[4]

The pathophysiology of IBS is still poorly elucidated, thereby hampering the development of effective therapies for IBS.[8] As one of the treatments in complementary and alternative medicine, acupuncture has been applied to gastrointestinal diseases in China for thousands of years, and its therapeutic effectiveness in IBS is being increasingly approved by more individuals.[9,10] However, the studies to date provided insufficient evidence to determine whether acupuncture is an effective treatment for IBS. A previous randomized controlled study concluded that “acupuncture in IBS is primarily a placebo response”.[11] Recent evidence[12] suggested that future RCTs should adhere to current Food and Drug Administration (FDA)-recommended composite endpoints for IBS which leads to lower placebo response rates. However, we did not find the use of them in clinical trials (especially for primary outcomes) of acupuncture for IBS, which may indicate a bias in the assessment of acupuncture efficacy in existing studies. Therefore, the first aim of the present study was to preliminarily test the feasibility of using FDA-recommended

endpoints to evaluate the efficacy of acupuncture in the treatment of IBS.

Furthermore, the acupoint is the key factor to determine the curative effect of acupuncture. Specific acupoints refer to the acupoints with special therapeutic function and naming in the meridians, which have specific treatment rules and wide applications. Comparing the therapeutic effects of different acupoint selection schemes is helpful to optimize the acupoint selection scheme and improve the clinical efficacy of acupuncture treatment. while the clinical efficacy among specific acupoints (SA), non-specific acupoints (NSA) and non-acupoints (NA) have not been revealed. Here, the second aim is to compare the difference in acupuncture efficacy in patients receiving a SA treatment, NSA treatment, or NA treatment.

4 Study hypotheses

The following hypotheses will be tested:

H1: There is a significant difference in the composite response rate at week 4 among the SA group, the NSA group and the NA group

H0: There is no difference in the composite response rate at week 4 among the SA group, the NSA group and the NA group

5 Methodology

5.1 Study design

This is a multicenter, randomized, controlled clinical trial. Patients with IBS will be randomly assigned to the SA group, the NSA group or the NA group in a 1:1:1 ratio.

5.2 Randomization and blinding

Sequence generation

Patients need to finish their defecation diary in the last 2 weeks before randomization. After screening, patients will be randomly assigned to SA group, NSA group and NA group according to the ratio of 1:1:1. The block randomization will be used in the trial. An independent statistician who is not involved in the implementation or statistical analysis of this trial will generate the blocked randomization sequence by using the software SAS 9.3.

Allocation concealment mechanism

The randomization sequence will be stored by the special randomization sequence manager and will not be available to other participating investigators. The allocation schedule will be using a telephone randomization procedure. The clinical research coordinators will be responsible for requesting randomization.

Implementation

The blocked randomization sequence will be generated by an independent statistician. The recruiter is responsible for registration. The acupuncturist will assign participants to related interventions.

Blinding

Patients, outcome assessor and statistician will be blinded to the assignments. During the treatment, the adhesive pads will be glued to the corresponding sterilized acupoints in order to make it impossible for patients to distinguish between the use of single-use sterile needles and blunt-tipped placebo needles. When two or more patients are treated at the same time, they will be screened or assigned to separate treatment rooms in order to refraining from communication. Interventions of patients will not be revealed until the statistical analysis is completed.

Every effort should be made to preserve the blind. Should a medical emergency arise that requires identification of the study acupuncture administered in order to manage the acute situation of the patient, the blind can be broken. The investigator should make every effort to contact the clinical

gastroenterologist to discuss the use of rescue medication or other necessary treatments. If blinding is broken ahead of time, it is necessary to record the time, reasons and executive personnel who break the blindness in advance, and notify the inspector as soon as possible. And related patients should not continue to participate in the trial, and the trial data can not be used for efficacy evaluation analysis, but should still be included in the safety analysis data set. Other available treatments should also be provided to related patients.

5.3 Sample size

This pilot study primarily aimed to preliminarily test the feasibility of using FDA-recommended endpoints to evaluate the efficacy of acupuncture in the treatment of IBS. The minimum sample size for exploratory trials is 20-30 per group according to Provisions for Drug Registration in China. Based on funding availability, we selected the maximum of 30 patients, and the sample size of 90 patients was determined. The results of this study will facilitate the calculation of an appropriate sample size for further randomized clinical trials.

5.4 Participant recruitment, screening and group assignment

Participants who are diagnosed as IBS-D according to the ROME IV will be recruited at (I) The First Affiliated Hospital of Hebei University of Chinese Medicine; (II) the Affiliated Hospital of Chengdu University of Traditional Chinese Medicine; (III) the Affiliated Hospital of Shandong University of Traditional Chinese Medicine; and (IV) the First Teaching Hospital of Tianjin University of Traditional Chinese Medicine. The recruitment strategy will primarily contain advertisements on outpatient clinics, and hospital social Internet media (WeChat). Written informed consent will be provided by each patient through research assistant before randomization. The evaluators will record the data on the electronic case report form (CRF) through the whole trial period. The participant flow was shown in Figure 1 and Figure 2.

	Baseline		Treatment Phase						Follow-up Phase	
Time Points (week)	Week -2	Week -1	Week 0	After first treatment	Week 1	Week 2	Week 3	Week 4	Week 8	Week 12
**Enrollment**										
Eligibility screen	×	×								
Informed consent	×	×								
Randomization			×							
**Interventions**										
SA				◆——	——	——	——	——◆		
NSA				◆——	——	——	——	——◆		
NA				◆——	——	——	——	——◆		
**Assessment**										
The response rate					×	×	×	×	×	×
IBS-SSS			×			×		×	×	×
PHQ-9			×			×		×	×	×
IBS-QOL			×			×		×	×	×
IBS-AR					×	×	×	×	×	×
Abdominal Pain Score	×	×			×	×	×	×	×	×
Abdominal Bloating Score	×	×			×	×	×	×	×	×
Bristol Stool Score	×	×			×	×	×	×	×	×
Blinding assessment								×		
Credibility and expectancy				×						
Rescue medicine					×	×	×	×	×	×
Adverse events				×	×	×	×	×	×	×

Figure 1 Procedure of treatment and visit

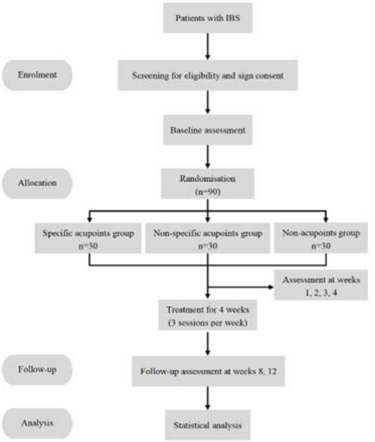

Figure 2 Flow diagram

5.5 Inclusion criteria

Participants will be included in the study if they meet the following criteria.

(I) Aged between 18 and 75 years (either sex);

(II) Diarrheal-predominant irritable bowel syndrome (IBS-D);

(III) Type 6 or 7 of the Bristol Stool Form Scale appeared for at least 4 days and type 1 or 2 appeared for less than 4 days in last 2 weeks; The average score of daily abdominal pain was ≥ 3 in the last week;

5.6 Exclusion criteria

Participants will be excluded from the study if they met the following criteria.

(I) Patients with the following diseases: inflammatory bowel disease, microscopic colitis, celiac disease, Crohn's disease and other organic bowel diseases; diabetes mellitus and abnormal thyroid function; severe acute or chronic organic diseases, kidney or liver diseases;

(II) History of previous abdominal surgery (appendectomy, hemorrhoidectomy, or polypectomy greater than 3 months post-surgery are allowed.);

(III) Pregnancy or lactation, or history of alcohol and drug abuse;

(IV) Treated with acupuncture in the last 6 months, or participating in other clinical trials;

(V) Usage of antidepressant or IBS medication within 2 weeks before treatment, including traditional Chinese medicine (TCM) or proprietary Chinese medicine, antidiarrheal, antispasmodic, intestinal antibiotics, probiotics and so on.

6 Outcome measurement

6.1 Primary outcome

In this trial, the response rate defined as the percentage of patients whose average value of worst abdominal pain is 30% better and the days of loose stool is 50% less than the baseline. The primary outcome is the response rate of week 4 after randomization. Patients will be asked to record daily IBS symptoms, including worst abdominal pain and stool type through Bristol Stool Scale. The score of worst abdominal pain in the preceding 24 hours will be evaluated by visual analogue scale (VAS) and average value of worst abdominal pain will be calculated weekly.

6.2 Secondary outcomes

The response rates at other time points: the response rate will also be measured at weeks 1, 2, 3, 8 and 12 after randomization.

IBS Symptom Severity Scale (IBS-SSS): The IBS-SSS contains five questions that are rated on VAS (0-100): severity of abdominal pain, frequency of abdominal pain, severity of abdominal distension, degree of dissatisfaction with defecation habits and interference with the quality of life. The above five aspects account for an equal proportion in the IBS-SSS with a score range of 0-500. A score below 175 indicates that the mild IBS syndrome, a score between 175 and 300 represents the moderate IBS syndrome, a score higher than 300 represents the severe IBS syndrome.[13] IBS-SSS will be used at weeks 2, 4, 8 and 12 after randomization.

Patient Health Questionnaire-9 depression scale (PHQ-9): PHQ-9 scores range from 0 to 27, by which depression is defined as mild (5-9), moderate (10-14), moderate (15-19) or severe (more than 20).[14] PHQ-9 will be used at weeks 2, 4, 8 and 12 after randomization.

IBS-Quality of Life scale (IBS-QOL): IBS-QOL, which consists of 34 items, will be used to assess the extent to which the quality of life of patients with IBS is disturbed. Each item will be evaluated with a 5-point Likert scale, and the cumulative total score of all items will be linearly converted into a 100-point scale. The higher score indicates the improvement in quality of life is more obvious.[15] IBS-QOL will be used at weeks 2, 4, 8 and 12 after randomization.

IBS Adequate Relief (IBS-AR): IBS-AR will be used to confirm whether the IBS symptoms of trial patients have been adequately relieved. This type of outcome has been shown to be associated with the improvement of individual symptoms[16] and has been extensively used to evaluate efficacy in IBS clinical trials.[17,18] The responder is defined as the patient who answered the question in the affirmative. IBS-AR will be used at weeks 1, 2, 3, 4, 8 and 12 after randomization.

Abdominal Pain Score: Patients will be asked to rate their worst abdominal pain in the past 24 hours. The pain will be recorded on VAS (0-10), where 0 corresponds to no pain and 10 corresponds to worst imaginable pain. The score will be measured at weeks 1, 2, 3, 4, 8 and 12 after randomization.

Abdominal Bloating Score: Patients will be asked to rate their worst abdominal bloating in the past 24 hours. The bloating will be recorded on VAS (0-10), where 0 corresponds to no bloating and 10 corresponds to worst imaginable bloating. The score will be measured at weeks 1, 2, 3, 4, 8 and 12 after randomization.

Bristol Stool Score (BBS): Patients will be asked to rate the BBS of the past 24 hours. The score is based on a 1 to 7 scale where 1 corresponds to a hard stool and 7 corresponds to watery diarrhea. And the frequency of each score will be also recorded. The BBS will be measured at weeks 1, 2, 3, 4, 8 and 12 after randomization.

Blinding assessment: To test whether blinding is successful, all patients will be asked to guess which kind of acupuncture they received at week 4 after randomization.

Credibility and expectancy: Credibility and expectancy will be evaluated using the Credibility/ Expectancy Questionnaire within 5 minutes after the first treatment.

Adverse events: All adverse events will be recorded during the whole treatment and follow-up phases. Based on the potential relationship between needling and adverse events, adverse events will be categorized as treatment-related or not.

7 Interventions

Before acupuncture treatment, licensed acupuncturists with at least three years of acupuncture experience will be trained in how to locate acupoints and non-acupoints, puncture and manipulate needles. All acupoints are localized according to the WHO Standard. Single-use sterile needles (0.30 mm in diameter and 30 mm in length or 0.30 mm in diameter and 25 mm in length; Hwato, Suzhou, China) will be used in SA group and NSA group. To help maximize blinding of patients in NA group, a blunt-tipped placebo needle will be used (similar to the Streitberger design)[19] which can provide patient-blinding effects with a similar appearance to conventional needles but no skin penetration.[20] And adhesive pads will be used in each group. Each patient will be scheduled to undergo 12 sessions of treatment, with each acupuncture lasting 30 minutes for 4 weeks (three sessions per week, every other day ideally). During the trial, patients will not be allowed to use other therapies or medications that have a therapeutic effect on IBS, such as traditional Chinese medicine (TCM) or proprietary Chinese medicine, antidiarrheal, antispasmodic, intestinal antibiotics, probiotics and so on. Loperamide (Imodium, Xian Janssen Pharmaceutical Ltd., China), will be used as rescue medication under the guidance of gastroenterologists whenever necessary. The medication status and other non-irritable bowel syndrome drugs applications of patients will be strictly recorded during the trial.

7.1 Specific acupoints group

The acupoints used in this group are composed of fixed acupoints and optional acupoints. Fixed acupoints include *Tianshu* (ST25), *Zhongwan* (RN12), *Guanyuan* (CV4), *Zusanli* (ST36), and *Shangjuxu* (ST37), which are commonly used in IBS patients. Optional acupoints will be selected based on the traditional Chinese acupuncture diagnosis of patients by the acupuncturist, such as *Taichong* (LR3) for syndrome of liver depression and spleen deficiency, *Sanyinjiao* (SP6) for syndrome of spleen deficiency and dampness obstruction, and *Neiting* (ST44) for syndrome of spleen-stomach damp-heat. After sterilization, the acupuncturist will insert single-use sterile needles into the deep tissue layers through adhesive pads of acupoints. Following needle insertion, small, equal manipulations of twirling, lifting,

and thrusting will be performed on all needles to reach *deqi* (a component sensation, including soreness, numbness, distension and heaviness). Acupuncture Locations and exhibited in Table 1.

Table 1 Locations of acupoints for SA group

	Acupoint	Location
Fixed acupoints of SA group	*Tianshu* (ST25)	On the horizontal line of the navel, 2 cun[a] beside the anterior midline
	Zhongwan (RN12)	On the anterior midline of upper abdomen, 4 cun superior to the navel
	Guanyuan (CV4)	On the anterior midline of abdomen, 3 cun inferior to the navel
	Zusanli (ST36)	3 cun directly below ST35, and one finger-breadth lateral to the anterior border of the tibia
	Shangjuxu (ST37)	On the anterolateral aspect of the leg, 6 cun inferior to the ST35, and one finger-breadth lateral to the anterior border of the tibia
Optional acupoints of SA group	*Taichong* (LR3)	In the depression anterior to the junction of the first and second metatarsal bones
	Sanyinjiao (SP6)	On the tibial aspect of the leg, posterior to the medial border of the tibia, 3 cun superior to the prominence of the medial malleolus
	Neiting (ST44)	On the instep, between the second and third toes of the red and white flesh behind the webbed margin

[a]1 cun (≈20 mm) is defined as the width of the interphalangeal joint of the patient's thumb

7.2 Non-specific acupoints group

The acupoints used in this group consist of six fixed acupoints (Table 2): *Shuifen* (RN9), *Liangmen* (ST21), *Yinjiao* (CV7), *Tiaokou* (ST38), *Yinshi* (ST33), *Lougu* (SP7). The rest of the operation is the same as that of SA group.

Table 2 Locations of acupoints for NSA group

	Acupoint	Location
Fixed acupoints of NSA group	*Shuifen* (RN9)	On the anterior midline of upper abdomen, 1 cun[a] superior to the navel
	Liangmen (ST21)	On the anterior midline of upper abdomen, 1 cun superior to the navel, 2 cun beside the anterior midline

Yinjiao (CV7)	On the anterior midline of abdomen, 1 cun inferior to the navel
Tiaokou (ST38)	On the anterolateral aspect of the leg, 8 cun inferior to the ST35, and on the line between ST35 and ST41
Yinshi (ST33)	On the anterior aspect of the thigh, 3 cun superior to basis patellae, and on the line between anterior superior iliac spine and basis patellae
Lougu (SP7)	On the anteromedial aspect of the leg and the tibial rear, on the line between medial malleolus and SP9, and 6 cun superior to medial malleolus

[a]1 cun (≈20 mm) is defined as the width of the interphalangeal joint of the patient's thumb

7.3 Non-acupoints group

Patients in this group will receive sham acupuncture with blunt-tipped placebo needles on non-acupoints. Five non-acupoints will be selected which are away from meridians or conventional acupoints. The use of blunt-tipped placebo needles will provide patients with the feeling of acupuncture but with no skin penetration and needle manipulation for *deqi*. The non-acupoints are shown in Table 3.

Table 3 Locations of acupoints for NA group

NA	Location
NA1	On the abdomen, 2 cun[a] superior to anterior superior iliac spine, between the gallbladder meridian and the spleen meridian
NA2	On the abdomen, 2 cun inferior to navel, 1 cun beside the anterior midline, between the kidney meridian and the stomach meridian
NA3	On the lateral aspect of the leg, 3 cun inferior to GB34, between gallbladder meridian and bladder meridian
NA4	On the leg, 2 cun superior to the medial malleolus, in the middle of the medial tibia, between the liver meridian and the spleen meridian
NA5	On the leg, the midpoint of the line between GB40 and ST41, between the gallbladder meridian and the stomach meridian

[a]1 cun (≈20 mm) is defined as the width of the interphalangeal joint of the patient's thumb

8 Data management and quality control

Before recruiting patients, the case report form (CRF) will be established to input and store data. The outcome evaluator will be responsible for recording the corresponding results of the outcome indicators in the appropriate location in the CRF. While the trial is completed, the data management team will lock the

database and the researchers can no longer modify the data. The patients' relevant information, name, ID card number and telephone number, will be kept anonymously. Some patients participate in the trial blindly and do not reasonably judge whether their own conditions can complete the content of the test, which will increase the drop-off rate. We have trained researchers to establish a good communication relationship with patients to ensure that patients have a more comprehensive understanding and cooperation of the trial before randomization. For patient who discontinue or deviate from intervention protocols, we will try to get as much trial data as possible by telephone, if patient is willing to provide it.

Documents of patients' information will be preserved for at least 5 years after publication. If readers and reviewers have any questions, in the meantime, they can contact the corresponding author for access to the original data. In addition, quality controllers will be independently established in each center to review and interpret the data of trial. They will review the progress of the trial, independently of the investigators, and decide whether the trial needs to be terminated early solely on the basis of adverse events.

Experts in acupuncture, gastroenterology, statistics and methodology reviewed and revised the trial protocol. Pre-specified standard operating procedures (intervention, details in filling CRF, results evaluation, data management) will be used for the training of related staff. All data modifications can be tracked through CRF. Appropriate communication will be maintained with the patients to strengthen their compliance. In addition, quality controllers will be set up in each branch center to control the quality of the research tasks undertaken by each branch center. Furthermore, the head research center inspector will monitor the trial process and data of each branch research center，when 10% and 90% of patients are included, respectively. If problems are found, the branch research center will be rectified and assessed in strict accordance with the relevant standard operating requirements of this trial. The trial will continue after the branch research center passes the assessment.

9 Statistical analysis

In this study, statisticians who blind to the group assignment will perform analyses using SPSS 22.0 statistical software. Continuous data will be represented by mean ± standard deviation (M ± SD) or median combined with interquartile range (IQRs), whereas categorical data will be represented by frequency, constituent ratio and percentage. Student's t test or Wilcoxon rank-sum test will be used for the comparison of continuous variables, and chi-square test or Wilcoxon rank-sum test will be used for the comparison of categorical variables. $P < 0.05$ is considered to indicate statistical significance. Intention-to-treat (ITT) set will be used in all efficacy analysis, which will consist of all patients who have been randomized into groups, and the missing outcome data will be complemented using last observation carried forward (LOCF) or multiple imputation.

For the primary outcome, covariate analysis will be conducted applying a logistic regression model that includes baseline covariates for both pain score and stool consistency. A per-protocol analysis will be used for primary outcome as sensitivity analysis covering patients who complete ⩾10 sessions and have no major protocol violations (taking other drugs during the trial, etc.).

10 Ethics and dissemination

The study protocol has been approved by the Medical Ethics Committee of Beijing University of Chinese. The randomized controlled trial has obtained the registration number (ChiCTR2000030670) and will be conducted in accordance with the rules of the Declaration of Helsinki. The recruiter in each branch center will be responsible for obtaining the informed consent of patients. Patients will be included only after the details of the study explained to them and signing informed consent forms. The results of

the trial will be published in a peer-reviewed academic journal.

References

1. Mearin F, Lacy BE, Chang L, et al. Bowel Disorders. *Gastroenterology*. 2016;S0016-5085(16)00222-5. doi:10.1053/j.gastro.2016.02.031

2. Longstreth GF, Thompson WG, Chey WD, et al. Functional bowel disorders. *Gastroenterology*. 2006;130(5):1480-1491. doi:10.1053/j.gastro.2005.11.06

3. Canavan C, West J, Card T. The epidemiology of irritable bowel syndrome. *Clin Epidemiol.* 2014;6:71-80. doi:10.2147/CLEP.S40245

4. Lovell RM, Ford AC. Global prevalence of and risk factors for irritable bowel syndrome: a meta-analysis. Clin. *Gastroenterol. Hepatol*. 2012;10(7):712-721.e4. doi:10.1016/j.cgh.2012.02.029

5. Maxion-Bergemann S, Thielecke F, Abel F, et al. Costs of irritable bowel syndrome in the UK and US. *Pharmacoeconomics*. 2006;24(1):21-37. doi:10.2165/00019053-200624010-00002

6. Sandler RS, Everhart JE, Donowitz M, et al. The burden of selected digestive diseases in the United States. *Gastroenterology.* 2002;122(5):1500-1511. doi:10.1053/gast.2002.32978

7. Gralnek IM, Hays RD, Kilbourne A, et al. The impact of irritable bowel syndrome on health-related quality of life. *Gastroenterology*. 2000;119(3):654-660. doi:10.1053/gast.2000.16484

8. Chang JY ,Talley NJ. Current and emerging therapies in irritable bowel syndrome: from pathophysiology to treatment. *Trends Pharmacol Sci*. 2010;31(7):326-334. doi:10.1016/j.tips.2010.04.008

9. Li H, He T, Xu Q, et al. Acupuncture and regulation of gastrointestinal function. *World J Gastroenterol.* 2015;21(27):8304-8313. doi:10.3748/wjg.v21.i27.8304

10. Ouyang H, Chen JDZ. Review article: therapeutic roles of acupuncture in functional gastrointestinal disorders. *Aliment Pharmacol Ther*. 2004;20(8):831-841. doi:10.1111/j.1365-2036.2004.02196.x

11. Schneider A, Enck P, Streitberger K, et al. Acupuncture treatment in irritable bowel syndrome. *Gut*. 2006;55(5):649-654. doi:10.1136/gut.2005.074518

12. Bosman M, Elsenbruch S, Corsetti M, et al. The placebo response rate in pharmacological trials in patients with irritable bowel syndrome: a systematic review and meta-analysis. *Lancet Gastroenterol Hepatol*. 2021;6(6):459-473. doi:10.1016/S2468-1253(21)00023-6

13. Francis CY, Morris J, Whorwell PJ, The irritable bowel severity scoring system: a simple method of monitoring irritable bowel syndrome and its progress. Aliment. Pharmacol. Ther. 1997;11:395-402.

14. Kroenke K, Spitzer RL, Williams JB. The PHQ-9: validity of a brief depression severity measure. *J Gen Intern Med*. 2001;16(9):606-613. doi:10.1046/j.1525-1497.2001.016009606.x

15. Patrick DL, Drossman DA, Frederick IO, et al. Quality of life in persons with irritable bowel syndrome: development and validation of a new measure. *Dig Dis Sci.* 1998;43(2):400-411.doi:10.1023/a:1018831127942

16. Mangel AW. Personal view: adequate relief as a primary endpoint in irritable bowel syndrome. *Aliment Pharmacol Ther*. 2006;23(7):879-881. doi:10.1111/j.1365-2036.2006.02839.x

17. Leventer SM, Raudibaugh K, Frissora CL, et al. Clinical trial: dextofisopam in the treatment of patients with diarrhoea-predominant or alternating irritable bowel syndrome. *Aliment Pharmacol Ther*. 2008;27(2):197-206. doi:10.1111/j.1365-2036.2007.03566.x

18. Camilleri M, Northcutt AR, Kong S, et al. Efficacy and safety of alosetron in women with irritable bowel syndrome: a randomised, placebo-controlled trial. *Lancet*. 2000;355(9209):1035-1040. doi:10.1016/S0140-6736(00)02033-X

19. Streitberger K, Kleinhenz J. Introducing a placebo needle into acupuncture research. *Lancet*.

1998;352(9125):364-365. doi:10.1016/S0140-6736(97)10471-8
20. Liu BY, Xu HF, Ma R, et al. Effect of blinding with a new pragmatic placebo needle: a randomized controlled crossover study. *Medicine (Baltimore)*. 2014;93(27):e200. doi:10.1097/MD.0000000000000200
