# Supplementary material for: Acupuncture for the Treatment of Diarrhea-Predominant Irritable Bowel Syndrome: A Pilot Randomized Clinical Trial
Source: JAMA Netw Open. 2022 Dec 29;5(12):e2248817. doi: 10.1001/jamanetworkopen.2022.48817 (PMC9856830; doi:10.1001/jamanetworkopen.2022.48817)
Supplement: Supplement 2. — eFigure 1. The Schematic Diagram of Sham-Acupuncture eFigure 2. Defecation Diary Card eFigure 3. The Procedure Diagram eFigure 4. Schematic Diagram of Changes in Each Outcome During Treatment and Follow-up eTable 1. Information on Patients Lost to Follow-up eTable 2. Distribution of the Completeness of the Composite Response Rate Data During Weeks 1-4 eTable 3. Blinding Assessment [file jamanetwopen-e2248817-s002.pdf]

Supplementary Online Content

Qi LY, Yang JW, Yan SY, et al. Acupuncture for the treatment of diarrhea-predominant irritable bowel syndrome: a pilot randomized clinical trial. *JAMA Netw Open*. 2022;5(12):e2248817. doi:10.1001/jamanetworkopen.2022.48817

eFigure 1. The Schematic Diagram of Sham-Acupuncture
eFigure 2. Defecation Diary Card
eFigure 3. The Procedure Diagram
eFigure 4. Schematic Diagram of Changes in Each Outcome During Treatment and Follow-up
eTable 1. Information on Patients Lost to Follow-up
eTable 2. Distribution of the Completeness of the Composite Response Rate Data During Weeks 1-4
eTable 3. Blinding Assessment

This supplementary material has been provided by the authors to give readers additional information about their work.

© 2022 Qi LY et al. *JAMA Network Open.*

eFigure 1. The Schematic Diagram of Sham-Acupuncture

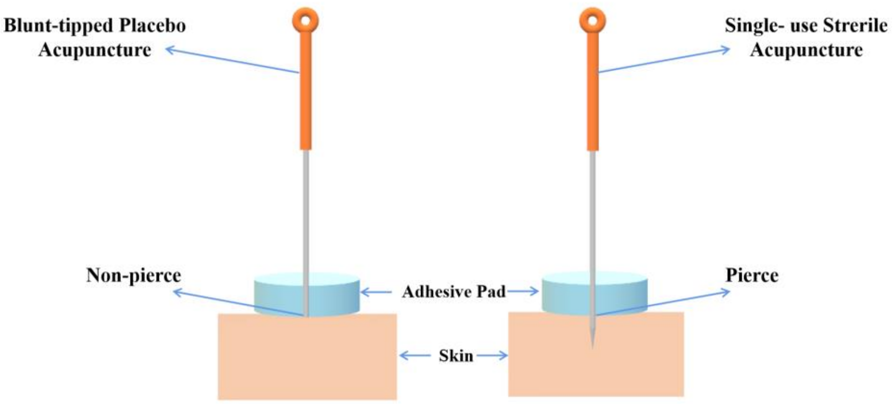

eFigure 2. Defecation Diary Card

Defecation diary card

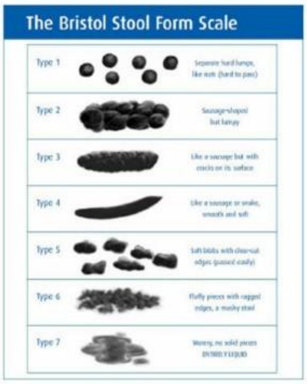

(Snapshots from ISBN 1-904156-08-8)

Type 1: 1 point

Type 2: 2 points

Type 3: 3 points

Type 4: 4 points

Type 5: 5 points

Type 6: 6 points

Type 7: 7 points

Name: __________

Contact number: ___________

Recording period: ☐ screening ☐ treatment ☐ follow-up

Record week: ☐☐ week

Recording time: ___________

1. Please record the number of bowel movements in the past 24 hours: (__) times
2. Please record the number of defecations with a sense of urgency in the past 24 hours: (__) times [cannot exceed the number of defecations entered before]
3. Please select the most representative stool type in the past 24 hours according to the Bristol Stool Scale: type (__)
4. Please draw a vertical line "|" on the corresponding position on the scale of the scale below according to the severity of the most severe abdominal pain in the past 24 hours:

Evaluate the degree of pain, 0 means no pain, 10 means unbearable severe pain
(no pain) 0 ——————— 10 (unbearable severe pain)

5. Please draw a vertical line "|" on the corresponding position of the scale below according to the most severe abdominal bloating in the past 24 hours:

Evaluate the degree of pain, 0 means no bloating, 10 means most severe bloating
(no bloating) 0 ——————— 10 (most severe bloating)

6. In the past 24 hours, how would you rate your irritable bowel syndrome symptoms? Please tick "√" before the corresponding level

☐ 0=None ☐ 1=Slight ☐2=Medium ☐ 3=Severe ☐ 4=Very serious

7. In the past 24 hours, have you taken emergency medicines (loperamide) or other medicines that have a therapeutic effect on irritable bowel syndrome? If so, please record the name of the medication, the time of medication, and the dosage in the form. [Note] If the number of medications exceeds 3 times, please record the rest of the medications in accordance with the table format.

Generic name	Usage	Dosage	Medication time

© 2022 Qi LY et al. *JAMA Network Open.*

eFigure 3. The Procedure Diagram

	Screening Phase			Treatment Phase						Follow-up Phase	
**Time Points (week)**	**Week -2**	**Week -1**	**Baseline**	**After first treatment**	**Week 1**	**Week 2**	**Week 3**	**Week 4**	**After last treatment**	**Week 8**	**Week 12**
**Enrollment**											
Eligibility screen	×	×									
Informed consent	×	×									
Defecation dairy	◆	—	—	—	—	—	—	—	—	—	◆
Randomization			×								
**Interventions**											
SA					◆	—	—	◆			
NSA					◆	—	—	◆			
NA					◆	—	—	◆			
**Assessment**											
The composite response rate					×	×	×	×		×	×
IBS-SSS			×			×		×		×	×
IBS-QOL			×			×		×		×	×
PHQ-9			×			×		×		×	×
IBS-AR					×	×	×	×		×	×
Abdominal pain	×	×			×	×	×	×		×	×
Abdominal bloating	×	×			×	×	×	×		×	×
Loose stool day	×	×			×	×	×	×		×	×
Stool frequency	×	×			×	×	×	×		×	×
Blinding assessment									×		
Credibility and expectancy				×							
Rescue medicine				◆	—	—	—	—	—	—	◆
Adverse events				◆	—	—	—	—	—	—	◆

© 2022 Qi LY et al. *JAMA Network Open.*

eFigure 4. Schematic Diagram of Changes in Each Outcome During Treatment and Follow-up

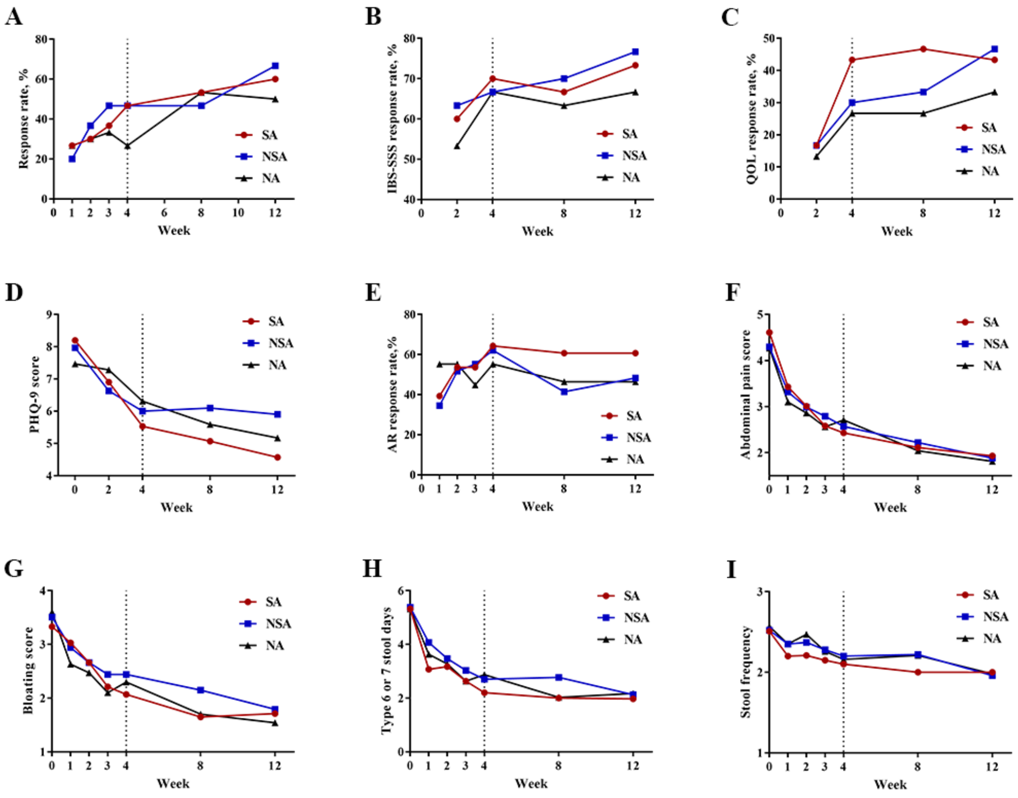

© 2022 Qi LY et al. *JAMA Network Open.*

eTable 1. Information on Patients Lost to Follow-up

	Trial phase		
**Group**	**4-week Treatment**	**8-week Follow-up**	**Total**
SA*	3 (10%)	1 (3.3%)	4 (13.3%)
NSA*	2 (6.7%)	1 (3.3%)	3 (13.3%)
NA*	3 (10%)	1 (3.3%)	4 (13.3%)

Note: SA = Specific Acupoint Group. NSA = Non-Specific Acupoint Group. NA = Non-Acupoint group.

*Data are provided with n (%). There was no difference among SA, NSA and NA in the number of lost to follow-up (P = 0.695, Fisher's exact test).

eTable 2. Distribution of the Completeness of the Composite Response Rate Data During Weeks 1-4

Patter N	Baseline	Week1	Week2	Week3	Week4	SA*	NSA*	NA*	Total
**1**	√	×	×	×	×	2 (6.7%)	1 (3.3%)	1 (3.3%)	4 (13.3%)
**2**	√	√	×	×	×	0	0	0	0
**3**	√	√	√	×	×	1 (3.3%)	0	2 (6.7%)	3 (10.0%)
**4**	√	√	√	√	×	0	1 (3.3%)	0	1 (3.3%)
**5**	√	√	√	√	√	26 (86.7%)	28 (93.3%)	27 (90%)	82 (91.1%)

Note: SA = Specific Acupoint Group. NSA = Non-Specific Acupoint Group. NA = Non-Acupoint group. √ = complete data. □ = incomplete data.

*Data are provided with n (%). Missing patterns were monotone and the difference among SA, NSA and NA on the missing patterns was not significant (P = 0.392, Fisher's exact test).

eTable 3. Blinding Assessment

Group	Acupuncture (n, %)	Sham acupuncture (n, %)	P
Acupuncture group(n=55)	20 (36.4)	35 (63.6)	0.050
Sham acupuncture group (n=27)	11 (40.7)	16 (59.3)	

Note: before the blinding assessment, the number of patients falling out of each group: SA=3, NSA=2, NA=3.

© 2022 Qi LY et al. *JAMA Network Open.*
