# Supplementary material for: Acupuncture for the Treatment of Diarrhea-Predominant Irritable Bowel Syndrome: A Pilot Randomized Clinical Trial
Source: JAMA Netw Open. 2022 Dec 29;5(12):e2248817. doi: 10.1001/jamanetworkopen.2022.48817 (PMC9856830; doi:10.1001/jamanetworkopen.2022.48817)
Supplement: Supplement 3. — Data Sharing Statement [file jamanetwopen-e2248817-s003.pdf]

Data Sharing Statement

Qi. Acupuncture for the Treatment of Diarrhea-Predominant Irritable Bowel Syndrome. *JAMA Netw Open*. Published December 29, 2022. doi:10.1001/jamanetworkopen.2022.48817

Data
Data available: Yes
Data types: Deidentified participant data, Data dictionary
How to access data: It will be made available to others upon request to the corresponding authors (lcz623780@126.com), only for research, and non-commercial purposes to individuals affiliated with academic or public health institutions.
When available: With publication

Supporting Documents
Document types: Statistical/analytic code, Informed consent form
How to access documents: It will be made available to others upon request to the corresponding authors (lcz623780@126.com), only for research, and non-commercial purposes to individuals affiliated with academic or public health institutions.
When available: With publication

Additional Information
Who can access the data: researchers whose proposed use of the data has been approved
Types of analyses: only for research
Mechanisms of data availability: with investigator support
